# Plagiarism awareness in post-graduate students: case study

**DOI:** 10.1038/s41598-026-56815-9

**Published:** 2026-06-14

**Authors:** Zivar Sabaghinejad, Parastoo Parsaei-Mohammadi, Narges Khanfari

**Affiliations:** 1 Knowledge & Information Science, Department of Medical Library and Information Science, School of Allied Medical Sciences, Ahvaz Jundishapur University of Medical Sciences, Ahvaz, Iran; 2 Department of Medical Library and Information Science, School of Allied Medical Sciences, Ahvaz Jundishapur University of Medical Sciences, Ahvaz, Iran

**Keywords:** Academic plagiarism, Understanding academic plagiarism, Writing skills, Citing skills, Check originality, Awareness of academic plagiarism, Education, Psychology, Psychology

## Abstract

Plagiarism in academic contexts presents a substantial ethical challenge within the field of publishing, impacting a diverse array of individuals, including faculty members and students. The present study employs a descriptive approach and was conducted using a survey method. The research sample consisted of 291 post-graduate students, selected through a stratified random sampling technique. The research instrument was a questionnaire. The collected data were analyzed using an independent samples t-test, and correlation coefficients. The collected data were analyzed using independent samples t-test, ANOVA, and correlation coefficients within the SPSS software environment (version 26). The findings demonstrated that the overall average awareness of academic plagiarism is higher among male participants than female participants; however, this difference was not statistically significant. Additionally, the average awareness of academic plagiarism in PhD students was significantly higher than Master’s students. The analysis revealed a significant difference in the average awareness of academic plagiarism among students from their first to fourth year, showing that awareness increased with each subsequent academic year. Furthermore, a positive and significant relationship was identified between the average awareness of academic plagiarism and academic level. The study revealed that as students progressed through their academic years, their awareness of the concepts and instances of academic plagiarism increased.

## Introduction

The term “academic plagiarism” was first introduced by Marcus Valerius to denote the misappropriation of intellectual achievements without the consent of the original authors^1^. According to Webster’s Dictionary^2^, plagiarism is defined as the improper use or theft of others’ words or ideas, done with the intention of creating new works without permission from the source. This act may be due to either ignorance or unfamiliarity with proper citation methods, or it may be deliberate to deceive others^3^. In this instance, the individual incorporates the writings of others into their new works without providing proper citation, thereby creating the impression that these writings are original^4^. This phenomenon and its consequences may involve various individuals, ranging from professors to students. Academic plagiarism among students can occur for severals reasons. For instance, it may stem from a desire for convenience, weaknesses in proper writing skills, time constraints^5^, and unfamiliarity with the concept and instances of academic plagiarism^6^.

Plagiarism among university students poses a significant threat to the integrity and effectiveness of educational institutions. This practice compromises the essential values of honesty and originality in academic pursuits by the unauthorized appropriation of others’ works, ideas, or expressions without appropriate acknowledgment. The widespread availability of information on the Internet further intensifies this issue, enabling students to easily incorporate plagiarized content with minimal effort. This behavior has been shown to impede students’ academic growth and hinder their ability to engage in critical thinking and idea development^7^. It also diminishes the perceived value of degrees and certifications conferred by educational institutions^8^. Addressing this issue requires a comprehensive strategy encompassing educating students about proper citation methods, implementing robust plagiarism detection systems, and fostering a culture of academic integrity^9^. Inadequate responses to plagiarism have the potential to erode the trust and credibility of the educational system, which can ultimately have a detrimental effect on the quality of education and the preparedness of graduates for the workforce.

Ajzen posits that an individual’s past experiences significantly inform their beliefs, which subsequently shape attitudes that exert influence on behavior^10^. The prevalence of academic plagiarism tends to be more pronounced at elevated educational strata, particularly within postgraduate programs^11^. An associated investigation indicated that 97.7% of postgraduate students are aware of academic plagiarism; however, 30% conceded engaging in plagiarism during the composition and dissemination of academic papers^12^. Likewise, an additional study revealed that 69.9% of pharmacy students had inadvertently perpetrated plagiarism^13^. These results underscore the disjunction between awareness and actual behavior. Various sociological and demographic variables, such as educational attainment, influence students’ cognizance of academic plagiarism; however, gender does not exert a considerable effect^14^. Research conducted in South India reported that approximately 20% of postgraduate students acknowledged having committed plagiarism, and the majority exhibited divergent attitudes towards plagiarism and its occurrences^15^. These findings accentuate the imperative to enhance awareness among students regarding the issue of academic plagiarism.

Chu, Li, and Mok^16^ assert that the issue of academic plagiarism among students is shaped by four key factors: “Understanding Plagiarism,” “Paraphrasing Skills,” “Citation Generation,” and “Originality Checking”. Their study introduces the UPCC model as a framework for analyzing instances of plagiarism. It is essential to grasp the definitions and consequences of plagiarism to foster ethical research practices, a topic examined through questions related to the “Understanding Plagiarism” aspect. The “Paraphrasing Skills” aspect evaluates students’ ability to rephrase ideas, thus enabling them to express their thoughts in their language and avoid direct copying. The “Citation Generation” aspect emphasizes the importance of developing skills and using appropriate tools for accurate citation, allowing students to effectively reference sources in their academic writing. Lastly, the “Originality Checking” aspect highlights students’ capabilities to verify the authenticity and reliability of the content they present.

A comprehensive search of the Scopus and Web of Science databases revealed approximately 14,000 articles about the subject of academic plagiarism (7,910 records in Scopus and 6,300 records in Web of Science). The upward trend in publications within this domain across both platforms suggests a growing concern among authors regarding publishing ethics. For postgraduate students, the obligation to complete theses while adhering to ethical standards to prevent plagiarism underscores the importance of research in this area. Moreover, with the increasing utilization of artificial intelligence tools in academic writing and the organization of research workshops on this subject, it is imperative to emphasize academic ethics and the recognition of plagiarism.

The present study aims to contribute to this body of knowledge by offering appropriate strategies to cultivate students’ adherence to publishing ethics. To this end, the study identifies the dimensions of academic plagiarism from the students’ perspective and describes the current situation.

The present study aims to contribute to this body of knowledge by offering appropriate strategies to cultivate students’ adherence to publishing ethics. To this end, the study identifies the dimensions of academic plagiarism from the students’ perspective and describes the current situation.

While previous studies have primarily focused on plagiarism awareness among medical students or undergraduate populations, this study specifically examines postgraduate students (Master’s and PhD) in a multidisciplinary Iranian university context. Furthermore, adopting the UPCC model^16^ for the first time in Iran, this research provides a comprehensive assessment across four dimensions: understanding, paraphrasing skills, citing skills, and originality checking. The findings offer practical insights for designing targeted educational interventions at different academic levels.

The primary aim of this study is to assess the level of plagiarism awareness among postgraduate students and to examine differences by gender, academic level, and academic year, using the UPCC model as a theoretical framework.

Accordingly, this study addresses the following research questions:

1. What is the level of awareness of academic plagiarism among postgraduate students (Master’s and PhD) at Ahvaz Jundishapur University of Medical Sciences (AJUMS)?
2. Is there a significant difference in plagiarism awareness based on gender and academic level (Master’s vs. PhD)?
3. How does plagiarism awareness vary across different academic years (first to fourth year)?
4. What is the relationship between the four components of the UPCC model (understanding, paraphrasing, citing, originality checking) and demographic variables?

## Method

This study adopts a quantitative, descriptive research design using a cross-sectional survey method^17^. The descriptive approach was chosen to systematically characterize the current status of plagiarism awareness among postgraduate students. The data collection instrument used in this study was a questionnaire, meticulously designed based on the seminal research contributions of Chu, Li, and Mok^16^ utilizing the UPCC model. The questionnaire comprises 31 questions, methodically organized into five sections: The first section, titled “Understanding Plagiarism” contained 10 questions. The second section, “Paraphrasing skills” comprised four questions. The third section, “Citing skills” consisted of five questions. The fourth section, “Originality Checking” included four questions. The final section, “Attitude toward Plagiarism” contained 12 questions. The questionnaire employed a six-point Likert scale, strongly disagree^1^, disagree^2^, somewhat disagree^3^, somewhat agree^4^, agree^5^, and strongly agree^6^.

To ensure cultural and linguistic appropriateness for the Iranian context, the questionnaire underwent a forward-backward translation procedure. First, the original English version was translated into Persian by two independent translators. A third translator then back-translated the Persian version into English, and any discrepancies were resolved through discussion among the research team.

Content validity was assessed by a panel of five experts (three faculty members in research methodology and two in academic ethics). They evaluated each item for relevance, clarity, and comprehensiveness. The Content Validity Index (CVI) was calculated, and items with a CVI below 0.78 were revised or removed. All final items met the acceptable threshold.

Face validity was confirmed through cognitive interviews with 10 postgraduate students (not included in the main sample), who confirmed that the questions were clear and understandable. Reliability was then evaluated through a pilot study with 50 postgraduate students from Ethics Committee of Ahvaz Jundishapur University of Medical Sciences (IR.AJUMS.REC.1401.162). Cronbach’s alpha coefficient for the entire questionnaire was 0.86, indicating good internal consistency.

The research population comprised postgraduate students from AJUMS in 2022 with the inclusion criteria being current enrolment and informed consent for participating in the study. The population was 1,194 students (953 Master’s students and 241 PhD students), and the sample size was determined using the Cochran formula to be 283 students. Subsequently, a simple stratified random sampling method was employed, resulting in a sample of 224 Master’s students and 59 PhD students. The normality of data distribution was assessed using the Kolmogorov-Smirnov test. The results were analyzed through frequency and percentage calculations, mean comparisons and group analyses employing t-tests and ANOVA within the SPSS software environment.

The data collection process for this study commenced after obtaining the ethics code. All methods were carried out in accordance with relevant guidelines and regulations. All experimental protocols were approved by the Ethics Committee of AJUMS (IR.AJUMS.REC.1401.162). Informed consent was obtained from all participants.

## Results

The demographic information of the research is presented in Table 1.

**Table 1:** Research demographic information.

Gender/educational level	Male	Female	MA	PhD
Frequency	85	198	224	59
Percentage	30	70	79.2	20.8

A substantial proportion of the research sample comprised of women, with the majority of the participants being Master’s students. In order to ascertain the normality of the data distribution, the Kolmogorov-Smirnov test was conducted for two independent groups. This test was performed separately for each item and component. The Z statistic, which yielded a value of 0.599 and a significance level of 0.866, indicated that the distribution was normal.

The objective of this study was to ascertain the plagiarism awareness status of post-graduate students regarding gender and academic level. To achieve this, an independent samples t-test was conducted, under the assumption of equal variances. The results of this test are presented in Table 2, which provides both the overall means and the means for each component related to plagiarism.

**Table 2:** Plagiarism awareness status in post-graduate students.

Component	Educational level		Gender	
	MA	PhD	Female	Male
Understanding Plagiarism	4.38	4.55	4.44	4.35
Paraphrasing skills	4.32	4.55	4.3	4.52
Citing skills	4.56	4.7	4.52	4.76
Originality Checking	4.23	4.4	4.22	4.37
Attitude	3.28	3.34	3.32	3.34
Total mean	4.1	4.24	4.12	4.15

As Table 2 indicates, the mean scores for all components, except for “attitude,” are higher for men than for women. Furthermore, the overall mean of plagiarism awareness is higher among PhD students than Master’s students, and higher among men compared to women. Subsequently, each component was compared using the independent samples t-test. Table 3 presents the findings of this test.

**Table 3:** Comparison of plagiarism awareness mean among post-graduate students.

Component	Variable	F	T	*P*-Value	Analyse
Understanding Plagiarism	Education level	1.87	1.6	0.11	The mean of PhD students is insignificantly higher than Ma.
	Gender	0.04	0.97	0.33	The mean of female is insignificantly higher than male.
Paraphrasing skills	Education level	0.6	0.07	0.26	The mean of PhD students is insignificantly higher than Ma.
	Gender	0.67	0.06	0.17	The mean of male is insignificantly higher than female.
Citing skills	Education level	0.56	1.16	0.24	The mean of PhD students is insignificantly higher than Ma.
	Gender	0.42	2.28	0.23	The mean of male is insignificantly higher than female.
Originality Checking	Education level	0.01	1.36	0.17	The mean of PhD students is insignificantly higher than Ma.
	Gender	1.06	1.38	0.16	The mean of male is insignificantly higher than female.
Attitude	Education level	1.58	0.61	0.54	The mean of PhD students is insignificantly higher than Ma.
	Gender	1.25	0.97	0.33	The mean of female is insignificantly higher than male.
Total Mean	Education level	0.02	2.29	0.02^*^	The mean of PhD students is significantly higher than Ma.
	Gender	0.51	0.65	0.51	The mean of male is insignificantly higher than female.

As shown in Table 3, the total mean of plagiarism awareness in all categories is higher than the statistical mean (3.5). Given these findings, attention to academic year could aid in better interpretation. Consequently, a one-way ANOVA test was conducted to compare the means by academic year. Academic year was defined as the year of study within the student’s current program (first through fourth year), combining both Master’s and PhD students. The test results, with a mean square of 0.428, an F-value of 2.326, and a significance level of 0.043, indicate a significant difference in plagiarism awareness among students in different academic years.

Subsequently, the LSD post hoc test was conducted to determine the nature of the differences between the groups (Table 4).

**Table 4:** Comparison total mean of plagiarism awareness based on academic years.

Comparison	Mean difference	*P*-value
1st year vs. 2nd year	0.15	0.01
1st year vs. 3rd year	0.15	0.03
1st year vs. 4th year	0.27	0.01

As shown in Table 4, post hoc comparisons using the LSD test indicated that first-year students had significantly lower plagiarism awareness compared to second-year students (mean difference = 0.15, *p* = 0.01), third-year students (mean difference = 0.15, *p* = 0.03), and fourth-year students (mean difference = 0.27, *p* = 0.01). No other pairwise comparisons reached statistical significance. These results suggest that plagiarism awareness increases significantly after the first year of postgraduate study and continues to rise through the fourth year.

To examine the relationships between the five components of plagiarism, gender, and academic level, Spearman’s correlation was utilized. The results of this examination are presented in Table 5.

**Table 5:** Correlation between plagiarism awareness components and demographic variables.

Variables	Component	Understanding Plagiarism	Paraphrasing skills	Citing skills	Originality Checking	Attitude	Total
Gender	Correlation coefficient	0.07	0.14	0.15	0.08	0.05	0.02
	P-value	0.2	0.08	0.008	0.17	0.35	0.66
Education level	Correlation coefficient	0.07	0.11	0.08	0.09	0.05	0.02
	P-value	0.02	0.006	0.01	0.09	0.05	0.01

An analysis of the correlation coefficient revealed no significant relationship between the mean of plagiarism awareness and gender. Conversely, a positive and significant correlation was observed between plagiarism awareness and academic level. This finding implies that as students advance in their academic journeys, their understanding of plagiarism improves. The notable relationship between plagiarism awareness and academic level, along with a significant difference in mean awareness levels among first-year students compared to their second, third, and fourth-year counterparts (as indicated by post hoc test results), prompted further correlation analysis between academic year and plagiarism awareness. The results demonstrated a positive and significant correlation, with a coefficient of 0.171 and a significance level of 0.004.

## Discussion

This study revealed that students’ understanding of plagiarism is above average, which is inconsistent with the findings of Jamshidi Boroujeni, Saeidi, and Heidari^19^ and Soheili, Azin, and Khasseh^20^. Factors associated with plagiarism awareness include Internet access, lack of self-efficacy in academic writing and research, societal moral values, cultural beliefs, inadequate university education, degree-oriented attitudes, overemphasis on grades, and psychological pressures^18^. Tremayne and Curtis^21^ concluded that individuals who understand and take plagiarism seriously are less likely to engage in it. The low level of awareness of plagiarism among students may be due to poor education about plagiarism concepts, lack of plagiarism-related content in relevant courses such as research methods, and inadequate training by relevant organizations such as universities. Therefore, it is crucial for university officials and administrators to use various methods, such as conducting workshops and training sessions, to raise students’ awareness about plagiarism and its consequences. Implementing comprehensive educational programs that focus on academic integrity can foster a culture of honesty and accountability among students, ultimately reducing plagiarism. By promoting a better understanding of proper citation practices and the importance of original work, institutions can empower students to take responsibility for their academic endeavors.This proactive approach not only enhances students’ academic skills, but also prepares them for ethical practices in their future careers, ensuring that they make positive contributions to their respective fields.

The findings of this study indicate that the average writing skills of PhD students are higher than those of Master’s students, although this difference is not statistically significant. In other words, no improvement in writing skills was observed with the increased academic level. Additionally, the comparison of this average between male and female groups did not show a significant difference. Krishmawan & Widiati^22^ demonstrated that students understand interpretation and paraphrasing as important tools to prevent plagiarism. They also found that many students face significant challenges in rephrasing sentences and conveying the original sentence’s meaning in a new sentence, often expressing an inability to do so. Sibomana, Ndayambaje and Uwambayinema^23^ identified proposed solutions to reduce plagiarism, such as teaching research methods, defining plagiarism and its examples, enhancing writing skills, and utilizing technology for plagiarism detection. Kargahi, Keshani, and Shah Siah^1^ noted that the primary cause of committing academic plagiarism is insufficient skills in scientific writing. The findings of this study align with previous research on the importance of writing skills. It can be concluded that writing skills have not been given significant emphasis in coursework and academic programs, which increases the likelihood of unintentional plagiarism. In addition, difficulties in writing in English are more pronounced for non-native English speakers^24^. This underscores the need for educational institutions to integrate comprehensive writing instruction into their curricula to ensure that all students develop the skills necessary to express their ideas clearly and ethically^25^. By prioritizing writing instruction, institutions can not only reduce instances of plagiarism, but also empower students to engage more deeply in their academic work and enhance their overall learning experience^26^. This approach fosters a culture of originality and critical thinking, equipping students with the tools to navigate complex ideas and articulate their thoughts effectively in both academic and professional settings. Research conducted by Ziaei and Zamani Bahabadi^27^ indicates that some students may inadvertently commit plagiarism owing to insufficient understanding of appropriate citation methods. In a related vein, Movahedian et al.^28^ demonstrated that enhancing students’ familiarity with citation techniques serves as a viable strategy for addressing ethical dilemmas and mitigating instances of plagiarism. It is essential to understand that paraphrasing skills in academic writing are vital for avoiding academic dishonesty. These skills enable students to articulate ideas in their own words while maintaining the original meaning. Good paraphrasing not only boosts authenticity but also encourages a deeper understanding and critical engagement with the sources. The development of cognitive thinking accompanies the learning of academic writing skills, as students need to grasp the text thoroughly to paraphrase it effectively, which in turn enhances their critical thinking and comprehension of the material^29^. Furthermore, utilizing text paraphrasing tools, particularly those powered by artificial intelligence, can significantly enhance students’ writing abilities and help them produce original academic work^30^.

The findings showed that PhD students tend to have a better grasp of how to control and review the authenticity of scientific work compared to Master’s students, with men performing better than women in this regard. Zamanzadeh and Ghafoori Fard^31^ argue that researchers’ awareness of scientific plagiarism, proper citation practices, and the use of plagiarism detection software can significantly help in preventing this type of misconduct. It is essential to provide training on how to effectively use plagiarism detection software to assess the authenticity of scientific work. Relying solely on students to analyze their content for scientific integrity and to prevent plagiarism using software is insufficient, underscoring the need for education in this area.

This research suggests that PhD students generally have more positive attitudes toward plagiarism than their Master’s peers, with female students showing more favorable views than male students. These attitudes are influenced by subjective norms, which are strengthened by the existence of clear and effective institutional policies. As students become more aware of the consequences of plagiarism, their perspectives change. A study by Zamani, Azimi, and Soleimani^32^ found that women typically have stronger deterrent attitudes towards plagiarism than men, which contrasts with the results of the current study. This difference may be attributed to variations in geographical contexts and the specific academic fields analyzed in the two studies. The current research encompasses faculties from the humanities, basic sciences, and engineering, while the earlier study was limited to a medical sciences university. Additionally, Abedini’s^33^ findings reveal a direct and positive relationship between attitudes towards plagiarism and both the frequency and severity of plagiarism incidents, as well as students’ academic performance. Therefore, it can be concluded that a more vigorous opposition to plagiarism correlates with a lower likelihood of its occurrence. The investigation by Ningtyas, Cahyono, and Khoiri^34^ demonstrated that students predominantly exhibited negative perceptions of plagiarism in the context of writing argumentative essays, especially concerning their behavioral, normative, and control beliefs. Notably, some students who articulated a more favorable perspective were referencing unintentional plagiarism rather than deliberate misconduct. In a related study, Nagii and John^35^ posited that students’ perceptions of plagiarism significantly shape their subjective norms that in turn influence their behavioral intentions. Their findings revealed a robust positive correlation between attitudes toward plagiarism and these subjective norms. Furthermore, research conducted by Javaid, Sultan, and Ehrich^36^ suggested that there exists only a marginal difference in the average attitudes toward plagiarism between first-year and final-year students, indicating that university initiatives aimed at combating plagiarism have not been particularly successful. As highlighted in various studies, fostering positive attitudes towards plagiarism is crucial for reducing its occurrence, and universities have a key role in encouraging these attitudes and enhancing students’ subjective norms.

An unexpected finding of this study is the notably lower mean score for the “Attitude toward Plagiarism” component (approximately 3.3 out of 6) compared to other components such as understanding (≈ 4.4) and citing skills (≈ 4.6). This discrepancy suggests that while postgraduate students possess adequate knowledge about what constitutes plagiarism and how to cite sources correctly, their personal attitudes toward plagiarism are less disapproving. Several explanations may account for this gap. First, students may experience intense pressure to publish and complete their theses within limited timeframes, leading to a pragmatic acceptance of certain plagiaristic practices. Second, repeated exposure to plagiarism in their academic environment without visible consequences may normalize the behavior, weakening attitudinal opposition. Third, the six-point Likert scale used in this study (without a neutral option) may have captured ambivalent responses as slightly positive. Future qualitative research is needed to explore the underlying reasons for this attitude-knowledge gap among Iranian postgraduate students.

The present investigation reveals that the average level of awareness regarding academic plagiarism surpasses the established statistical norm. PhD students exhibit a greater overall understanding of plagiarism than master’s counterparts, while male students demonstrate a higher level of awareness than female students. This finding implies that as students advance through their academic journeys, their comprehension of plagiarism tends to enhance. An analysis of awareness across different academic years indicates that first-year students exhibit a significantly lower awareness of plagiarism than second, third, and fourth-year students. Research conducted by Elliot and Brown in 2024 further supports this observation, indicating that first-year students have a limited grasp of plagiarism and its various manifestations. Pournaqi and Khosravi^37^ have also reported that students’ awareness of plagiarism is moderate. A notable correlation exists between students’ awareness of plagiarism and their ethical perceptions. Ligi and Trasberg^38^ identified that many students lack awareness regarding plagiarism regulations and face personal difficulties, such as effectively summarizing critical information, which significantly contributes to the prevalence of plagiarism. Engkizar and his research team^39^ identified eight primary factors contributing to plagiarism in their investigation. These factors encompass a lack of understanding of plagiarism, student`s commitment to academic responsibilities, an excessive volume of academic assignments, disinterest in reading, time constraints, easy access to technology, limited financial means, and inadequate knowledge of scientific writing. Additionally, Clarke and his associates^40^ observed that undergraduate students demonstrated a lower ability to identify instances of plagiarism compared to their peers at the master’s level. Research demonstrates that the recognition of academic plagiarism tends to increase as individuals attain higher educational levels. This increase is primarily attributed to students’ commitment to producing acceptable research as they progress toward their degrees. To facilitate this understanding, it is crucial to impart essential skills. Various strategies can be employed: presenting students with instances of plagiarism to enhance their understanding and attitudes; conducting workshops aimed at developing writing and revision skills to bolster their writing proficiency; providing training sessions on citation practices to underscore the importance of accurate citation in producing high-quality scholarly work; and introducing students to plagiarism detection tools to raise their awareness and assist in preventing plagiarism.

The non-significant gender difference in plagiarism awareness observed in this study aligns with some previous research (Nketsiah et al., 2023) but contradicts others (Zamani et al., 2013). One possible explanation is that the postgraduate environment at AJUMS provides relatively uniform academic training and access to resources for both male and female students, reducing gender-based disparities in awareness. Additionally, the predominantly female sample (70%) may have limited statistical power to detect small gender effects. However, it is important to note that this study did not control for potential confounding variables such as English language proficiency, prior participation in plagiarism workshops, or disciplinary background. Students with higher English proficiency, for instance, may have better access to international guidelines on academic integrity. Similarly, students in humanities and social sciences may receive different training on citation practices compared to those in basic sciences or engineering. Future research should include these variables to better isolate the net effect of gender on plagiarism awareness.

A limitation of this study is the possibility of inaccuracies in the responses gathered through the questionnaire. Furthermore, as the study sample comprised graduate students from a single university, the findings may not be generalizable to all student demographics or other institutional contexts. Additionally, the cross-sectional design precludes causal inferences about the development of plagiarism awareness over time. Furthermore, this study did not control for potential confounding variables such as English language proficiency, prior training in academic writing or plagiarism detection, or field of study. These factors may independently influence plagiarism awareness and should be included in future investigations.

## Conclusion

As students progress in their academic pursuits, their awareness of plagiarism increases significantly. With extended exposure to academic environments and the refinement of their writing and summarization skills, they become more adept at avoiding instances of plagiarism. This is particularly crucial for graduate students, who must acknowledge that replicating another individual’s work poses a serious ethical dilemma, often exacerbated by the substantial pressure to deliver high-quality research papers within stringent time constraints. Such actions are entirely unacceptable. The integrity of scholarly work is jeopardized by direct replication, poorly structured paragraphs, or improper citations. The primary factors contributing to plagiarism include a lack of comprehension regarding appropriate citation methods, insufficient writing proficiency, and the intense pressure to publish. In response to these challenges, higher education institutions utilize plagiarism detection software and offer training on academic integrity, thereby assisting students in improving their writing skills while cultivating a culture that emphasizes innovation and ethical research practices.

However, several limitations should be acknowledged. First, the data were self-reported, which may introduce social desirability bias. Second, the sample was limited to a single university, limiting generalizability to other contexts. Third, the cross-sectional design precludes causal inferences.

Future research should employ longitudinal designs to track changes in plagiarism awareness over time, include students from multiple universities across Iran, and incorporate qualitative interviews to explore the reasons behind low paraphrasing and citation skills. Additionally, intervention studies are needed to evaluate the effectiveness of workshops and plagiarism detection software training.

## Data Availability

The data has been collected actively for the study. The datasets used and analysed during the current study available from the corresponding author on reasonable request.
